# Point-of-Care Technologies for Health Care

**DOI:** 10.1109/TBME.2011.2109251

**Published:** 2011-03-10

**Authors:** Fred R. Beyette, Charlotte A. Gaydos, Gerald J. Kost, Bernhard H. Weigl

**Affiliations:** 1 School of Electronic and Computing SystemsUniversity of Cincinnati Cincinnati OH 45221 USA; 2 Div. Infectious Disease, MedicineJohn Hopkins University Baltimore MD 21205 USA; 3 Pathology and Laboratory MedicineSchool of MedicineUniversity of California Davis CA 95616 USA; 4 Diagnostics, PATHBioEUniversity of Washington Seattle WA 98121 USA

## Abstract

The increasingly global focus on health care issues continues to underline the importance of point-of-care technologies and their ability to provide cost-effective solutions that address many unmet health care needs. Further, the current crisis in health care costs has critically underscored the need for research and development into highly effective, but low cost means of delivering health care. With a focus on providing clinically actionable information at or near the patient, point-of-care devices provide clinicians with information that is critical to the management of patient care while they are still with the patient. Rapid information results in various advantages for POC testing in different kinds of health care settings. In primary care settings in developed countries, the shortened timeline between testing and availability of results reduces the need for extra office visits or follow-up phone calls to convey testing results and adjust clinical intervention. This strategy can reduce cost and increase access of otherwise underserved populations to medical care. For diseases that are infectious, such as sexually transmitted infections or respiratory diseases, POC testing can facilitate treatment modalities quickly, thus preventing further spread of the infection for better and timely clinical management. In acute care settings, timely access to diagnostic information is most critical for providing an effective medical response. In disaster settings, POC diagnostics can speed triage and enable rapid establishment and delivery of medical services.

The increasingly global focus on health care issues continues to underline the importance of point-of-care technologies and their ability to provide cost-effective solutions that address many unmet health care needs. Further, the current crisis in health care costs has critically underscored the need for research and development into highly effective, but low cost means of delivering health care. With a focus on providing clinically actionable information at or near the patient, point-of-care devices provide clinicians with information that is critical to the management of patient care while they are still with the patient. Rapid information results in various advantages for POC testing in different kinds of health care settings. In primary care settings in developed countries, the shortened timeline between testing and availability of results reduces the need for extra office visits or follow-up phone calls to convey testing results and adjust clinical intervention. This strategy can reduce cost and increase access of otherwise underserved populations to medical care. For diseases that are infectious, such as sexually transmitted infections or respiratory diseases, POC testing can facilitate treatment modalities quickly, thus preventing further spread of the infection for better and timely clinical management. In acute care settings, timely access to diagnostic information is most critical for providing an effective medical response. In disaster settings, POC diagnostics can speed triage and enable rapid establishment and delivery of medical services.

While in the developed world, POC testing is primarily designed as an adjunct to central lab testing, not as replacement, POC testing can enable local health care providers to deliver cost-effective care in developing countries or at rural locations with lack of access to central laboratory infrastructure. Further, a growing number of point-of-care technologies enable clinicians to remotely assess/monitor patients who are home bound or unable to meet the clinician in their clinical setting. The underlying theme is to multiply the effectiveness of physicians by providing better/faster information that enables timely delivery and management of health care.

## I. Scope of the Special Issue

For this Special Issue of IEEE Transactions on Biomedical Engineering (TBME) LETTERS, we invited manuscripts highlighting highly innovative, novel, and exciting research activities in wearable sensors, remote health monitoring, e-Health, telemedicine, and health care information management for acute emergency care, clinical disease, and outbreak/disaster situations. As a result of the call, we received a record number of 73 submissions. These manuscripts were submitted by leading experts in their respective areas. From the many meritorious submissions, 24 manuscripts with high potential impact were selected to present a wide cross-section of the growing diversity of clinical needs where point-of-care technologies show a significant promise to improve the delivery of health care.

## II. Special Issue Papers

The papers in this Special Issue highlight some of the most exciting current research in the development of novel point-of-care technologies. Covering clinical applications that range from point-of-care testing for Chlamydia to remote monitoring of cardiac condition, the papers in this issue provide a snap shot of the broad range of unmet clinical needs that are being addressed at the point of need.

The papers have been loosely divided into two groups. Twelve papers highlighting advances in Sensors and Devices focus on advancements that improve aspects of the core technologies that underpin various point-of-care technologies. The second group of twelve papers focuses on Detection, Analysis and Monitoring in various clinical needs.

The following sections provide a brief highlight for each of the articles that make up this Special Issue on Emerging Technologies in Point-of-Care Health Care.

### A. Sensors and Device

Linghua *et al.* describe a novel instrument for multispectral imaging. Their handheld device provides real-time operation at a cost that is suitable for home-based health care applications.

Jung *et al.* present a handheld point-of-care device for optical coherence tomography. The system provides a user-friendly interface that is capable of guiding the physician in real-time toward the identification of suspicious tissue regions that needed closer examination.

Motivated by the disparity in cardiac care between rural and urban health care delivery, Mandal *et al.* developed a point-of-care device specifically intended for delivery of heart care services to rural populations. Preliminary results confirm the applicability of the device as a pre-screening tool that provides indicative diagnosis of cardiac conditions.

D'Arcy *et al.* describe a noninvasive device that measured brainwaves (electroencephalography or EEG). In contrast to traditional EEG systems, their device is portable, user-friendly and includes a novel software algorithm that automates stimulation, data acquisition/analysis and reporting functions to enable evaluation of conscious awareness in brain damaged patients.

Pearce *et al.* have developed a Point-of-Care device for detection of Chlamydia trachomatis. Utilizing a novel electrochemical detection method, they are able to run their assay in less than 25 minutes while maintaining sensitivity and specificity of 98%.

Bercich *et al.* present a hand held plasma isolation device that utilizes a unique array of parallel fiber glass filters to produce a plasma sample from a small quantity of whole blood. Their device is a critical enabling technology in the development of Point-of-Care technologies that analyze plasma.

Majerus *et al.* describe the design, fabrication and testing of a wireless bladder pressure sensor. The device is intended for chronic, ambulatory application, such as urodynmics or closed-loop neuromodulation.

Gao details the design of an integrated CMOS untra-wideband wireless telemetry transceiver for wearable and implantable medical sensor applications. A prototype implementation of the transceiver is then demonstrated in a capsule endoscopy device that is capable of in vivo transmission of 640 × 480 resolution images at a frame rate of 2.5 frames/second.

Beyette *et al.* demonstrate the performance of a device that quantifies bilirubin in hemorrhagic CSF. By identifying bilirubin even in the presence of hemoglobin, the device is able to differentiate bleeding associated with a subarachnoid hemorrhage from bleeding that is associated with a traumatic spinal tap.

Using diffuse reflectance spectroscopy, Alla *et al.* present results for a device that can noninvasively quantify bilirubin, hemoglobin, and hematocrit. The device is tested using an animal model for jaundice and human subjects.

Geddes *et al.* show their microwave accelerated metal enhanced fluorescence method for lysing Chlamydia trachomatis and then detecting the DNA released from the lysed cells. Their new “release and detect” method allows for detection of bacteria in less than 1 min.

Jablónski describes work done to develop a mobile interrupter module for monitoring respiratory mechanics. Especially well suited for newborns, preschool children, and patients suffering from respiratory muscle impairment, this enhanced interrupter technology is well suited for home-based monitoring in telemedicine applications.

### B. Detection, Analysis, and Monitoring

Chon and Lee present a particle filtering algorithm that combines both time variant and time invariant autoregressive models for accurate extraction of respiratory rates. The method is able to accurately measure respiration rates in the range from 6 to 90 bpm even when the respiration rate suddenly changed (both increase and decrease) by 24 bpm.

Giancardo *et al.* propose a technique that uses uncalibrated multiple-view fundus images to analyze swelling of the macula. The reconstructed images resulting from there method may enhance the performance of fundus cameras that are used in the diagnosis of various retinal diseases.

Pecchia *et al.* describe a platform to enhance effectiveness and efficiency of home monitoring through a method of data mining. The system is focused on early detection of indicators that a patient's condition is worsening.

Bearinger *et al.* present the development of a small footprint, disposable, fast and inexpensive device for pathogen detection. Utilizing a variation of Loop-Mediated Isothermal Amplification (LAMP) assay, their system provides a screening tool with PCR level sensitivity and specificity.

Youn *et al.* discuss a sensor integrated system model for metabolic syndrome prediction. Using their system, they show the possibility to evaluate the functionality of human mitochondria and analyze energy metabolism.

Barfield *et al.* report the development of a new lateral flow rapid test for Chagas disease. With sensitivity/specificity results that are comparable to reference tests, their new method shows promise as an improved and reliable tool for screening and diagnosis of Chagas disease.

Ruggeri *et al.* describe a novel system for vascular tree identification and the quantitative estimation of AVR clinical index in retinal fundus images. The system is organized as a client-server structure that enables utilization by clinicians and researchers from all over the world.

Poh *et al.* present a simple, low-cost method for measuring several physiologic parameters using a webcam. Their system, which quantifies blood volume pulse, heart rate, respiratory rate, and heart rate variability, has significant potential for advancing personal health care and telemedicine. This letter was intended to be part of the Special Issue, but was previously published in the IEEE Transactions on Biomedical Engineering, January 2011 issue.

Lahuec *et al.* propose a new metric for in vivo estimation of the lifespan for total knee replacement prosthesis. In contrast to current methods, which cannot be applied until years after knee replacement surgery, their method enables lifespan estimation of the prosthesis a couple of months after surgery.

Rico *et al.* report on a microfluidic chip-based hydrodynamic focusing approach that minimizes sample volumes required in the analysis of cell-surface interactions. Their system quantifies cellular surface coverage and aggregate size distributions as a function of time during blood-flow analysis and facilitates diagnosis of disease state and/or efficacy of drug treatment.

Bonato *et al.* describe a platform to enable home monitoring of patients with Parkinson's disease using wearable sensors. Performance analysis of the system suggests that their web-based technology is suitable for facilitating the titration of medications for patients in the late stages of the disease.

Hesse *et al.* evaluate the feasibility and performance of a new Point-of-Care test for detection of Chlamydia trachomatis. Rapid communication with the device manufacture of results from a small study that analyzed self-test utilization of the device compared to the gold standard physician collected cervical tests enabled rapid turnaround in the design modification.

